# Appropriate referral and selection of patients with chronic pain for spinal cord stimulation: European consensus recommendations and e‐health tool

**DOI:** 10.1002/ejp.1562

**Published:** 2020-04-04

**Authors:** Simon Thomson, Frank Huygen, Simon Prangnell, José De Andrés, Ganesan Baranidharan, Hayat Belaïd, Neil Berry, Bart Billet, Jan Cooil, Giuliano De Carolis, Laura Demartini, Sam Eldabe, Kliment Gatzinsky, Jan W. Kallewaard, Kaare Meier, Mery Paroli, Angela Stark, Matthias Winkelmüller, Herman Stoevelaar

**Affiliations:** ^1^ Department of Anaesthesiology Basildon and Thurrock University Hospitals Basildon UK; ^2^ Department of Anaesthesiology Erasmus University Medical Center Rotterdam The Netherlands; ^3^ Clinical Neuropsychology Service Oxford University Hospitals Oxford UK; ^4^ Valencia University Medical School Anesthesia Unit – Surgical Specialties Department, Department of Anaesthesiology, Critical Care and Pain Management General University Hospital Valencia Spain; ^5^ Leeds Pain and Neuromodulation Centre Leeds Teaching Hospitals Leeds UK; ^6^ Department of Neurosurgery Fondation Ophtalmologique Adolphe de Rothschild Paris France; ^7^ Neuromodulation Team Wessex Neurological Centre Southampton UK; ^8^ Department of Anaesthesiology AZ Delta Roeselare Belgium; ^9^ Department of Physiotherapy Basildon and Thurrock University Hospitals Basildon UK; ^10^ Anaesthesiology & Pain Therapy Unit Santa Chiara University Hospital Pisa Italy; ^11^ Pain Unit Clinical Scientific Institutes Maugeri Pavia Italy; ^12^ Department of Pain Medicine The James Cook University Hospital Middlesbrough UK; ^13^ Department of Neurosurgery Sahlgrenska University Hospital Gothenburg Sweden; ^14^ Department of Anaesthesiology and Pain Management Rijnstate Hospital Velp The Netherlands; ^15^ Department of Neurosurgery and Department of Anaesthesiology Aarhus University Hospital Aarhus Denmark; ^16^ Pain Management Service Basildon and Thurrock University Hospitals Basildon UK; ^17^ Department of Neurosurgery Friederikenstift Hannover Hannover Germany; ^18^ Centre for Decision Analysis and Support Ismar Healthcare Lier Belgium

## Abstract

**Background:**

Spinal cord stimulation (SCS) is an established treatment for chronic neuropathic, neuropathic‐like and ischaemic pain. However, the heterogeneity of patients in daily clinical practice makes it often challenging to determine which patients are eligible for this treatment, resulting in undesirable practice variations. This study aimed to establish patient‐specific recommendations for referral and selection of SCS in chronic pain.

**Methods:**

A multidisciplinary European panel used the RAND/UCLA Appropriateness Method (RUAM) to assess the appropriateness of (referral for) SCS for 386 clinical scenarios in four pain areas: chronic low back pain and/or leg pain, complex regional pain syndrome, neuropathic pain syndromes and ischaemic pain syndromes. In addition, the panel identified a set of psychosocial factors that are relevant to the decision for SCS treatment.

**Results:**

Appropriateness of SCS was strongly determined by the neuropathic or neuropathic‐like pain component, location and spread of pain, anatomic abnormalities and previous response to therapies targeting pain processing (e.g. nerve block). Psychosocial factors considered relevant for SCS selection were as follows: lack of engagement, dysfunctional coping, unrealistic expectations, inadequate daily activity level, problematic social support, secondary gain, psychological distress and unwillingness to reduce high‐dose opioids. An educational e‐health tool was developed that combines clinical and psychosocial factors into an advice on referral/selection for SCS.

**Conclusions:**

The RUAM was useful to establish a consensus on patient‐specific criteria for referral/selection for SCS in chronic pain. The e‐health tool may help physicians learn to apply an integrated approach of clinical and psychosocial factors.

**Significance:**

Determining the eligibility of SCS in patients with chronic pain requires careful consideration of a variety of clinical and psychosocial factors. Using a systematic approach to combine evidence from clinical studies and expert opinion, a multidisciplinary European expert panel developed detailed recommendations to support appropriate referral and selection for SCS in chronic pain. These recommendations are available as an educational e‐health tool (https://www.scstool.org/).

## 1 INTRODUCTION

It is estimated that chronic pain affects around 20% of the adult population in developed nations (Breivik, Collett, Ventafridda, Cohen, & Gallacher, 2006; Dahlhamer et al., 2018). The management of chronic pain is often complex and may consist of pharmacological therapy, surgery, minimally invasive treatments, physiotherapy, psychological and behavioural treatments, or combinations thereof (Verrills, Sinclair, & Barnard, 2016). Within this therapeutic arsenal, spinal cord stimulation (SCS) has gained an established position for selected patients.

There is substantial evidence from randomized controlled trials (RCTs) on the efficacy of SCS in patients with persisting pain after previous spine surgery (De Andres et al., 2017; Kapural et al., 2015; Kumar et al., 2007; North, Kidd, Farrokhi, & Piantadosi, 2005), in patients with complex regional pain syndrome (CRPS) (Kemler et al., 2000) and in patients with painful diabetic neuropathy (van Beek et al., 2018; Slangen et al., 2014; de Vos et al., 2014). For other neuropathic pain syndromes (NPS), such as postherpetic neuralgia and phantom and stump pain, case reports and single‐arm studies report positive effects of SCS, but controlled studies are needed to confirm these findings (Corbett et al., 2018; Kurklinksy, Palmer, Arroliga, & Ghazi, 2018). RCTs have shown that SCS may also be beneficial in ischaemic conditions such as refractory angina pectoris (Pan et al., 2017) and critical limb ischaemia (Ubbink & Vermeulen, 2013). However, data on the specific effect of SCS on the pain component of these conditions are less conclusive as these studies typically use other (primary) outcome measures (e.g. angina frequency, limb salvage).

The outcome of SCS in patients with chronic pain may be negatively affected by several factors including substance abuse, psychological distress and mental health problems (De La Cruz et al., 2015; Fama et al., 2016). As these problems may often be reversible to an acceptable level, adequate identification and appropriate management, preferably in a multidisciplinary setting, are crucial.

The complex interaction of clinical and psychosocial factors that determine the eligibility of patients with chronic neuropathic, neuropathic‐like and ischaemic pain for the consideration of SCS has led to a situation in which selection criteria are usually opaque for referrers, and often lack consistency among implant centres. The available guidelines (a.o. Cruccu et al., 2016; Dworkin et al., 2013; National Institute for Health & Clinical Excellence, 2008) are not very explicit in their recommendations and certainly do not address the heterogeneity of patients seen in daily clinical practice.

This study aimed firstly at establishing patient‐specific criteria for the appropriate referral and selection for SCS in chronic pain based on the best available evidence from clinical studies and the expertise of a European multidisciplinary expert panel. In addition, we aimed at developing an educational e‐health tool based on the panel outcomes that allows both referrers and implanters to learn about the appropriate (pre)selection of patients with chronic pain for the consideration of SCS, with the eventual goal of improving patient outcomes.

## 2 METHODS

To explore the indications for SCS in patients with chronic pain, we used the RAND/UCLA Appropriateness Method (RUAM) (Brook et al., 1986). This modified Delphi method aims at systematically combining evidence from clinical studies and expert opinion to establish patient‐specific recommendations on the appropriateness of medical, surgical and diagnostic procedures. The RUAM has been applied in various disease areas, and has been extensively tested for its reliability and predictive validity (Lawson, Gibbons, Ko, & Shekelle, 2012). It is particularly helpful if data from clinical studies are insufficient to address the heterogeneity of patients in daily clinical practice (Fitch et al., 2001).

### 2.1 Panel composition

We composed a European expert panel representing the disciplines that are commonly involved in the evaluation of patients for SCS (anaesthesiology, neurosurgery, psychology, physiotherapy and nursing). Selection of panel members was based on their scientific and/or clinical expertise in the field of SCS. The panel consisted of 18 experts from 9 European countries (see supporting information).

### 2.2 Literature study

A literature study was conducted to establish an overview of the evidence from clinical studies on the efficacy and safety of SCS for different types of chronic pain, as well as on predictive factors for success or failure. The study results were used to shape the study design and to ensure that panellists had access to the same body of evidence while doing the ratings.

### 2.3 Panel process

**FIGURE 1:**
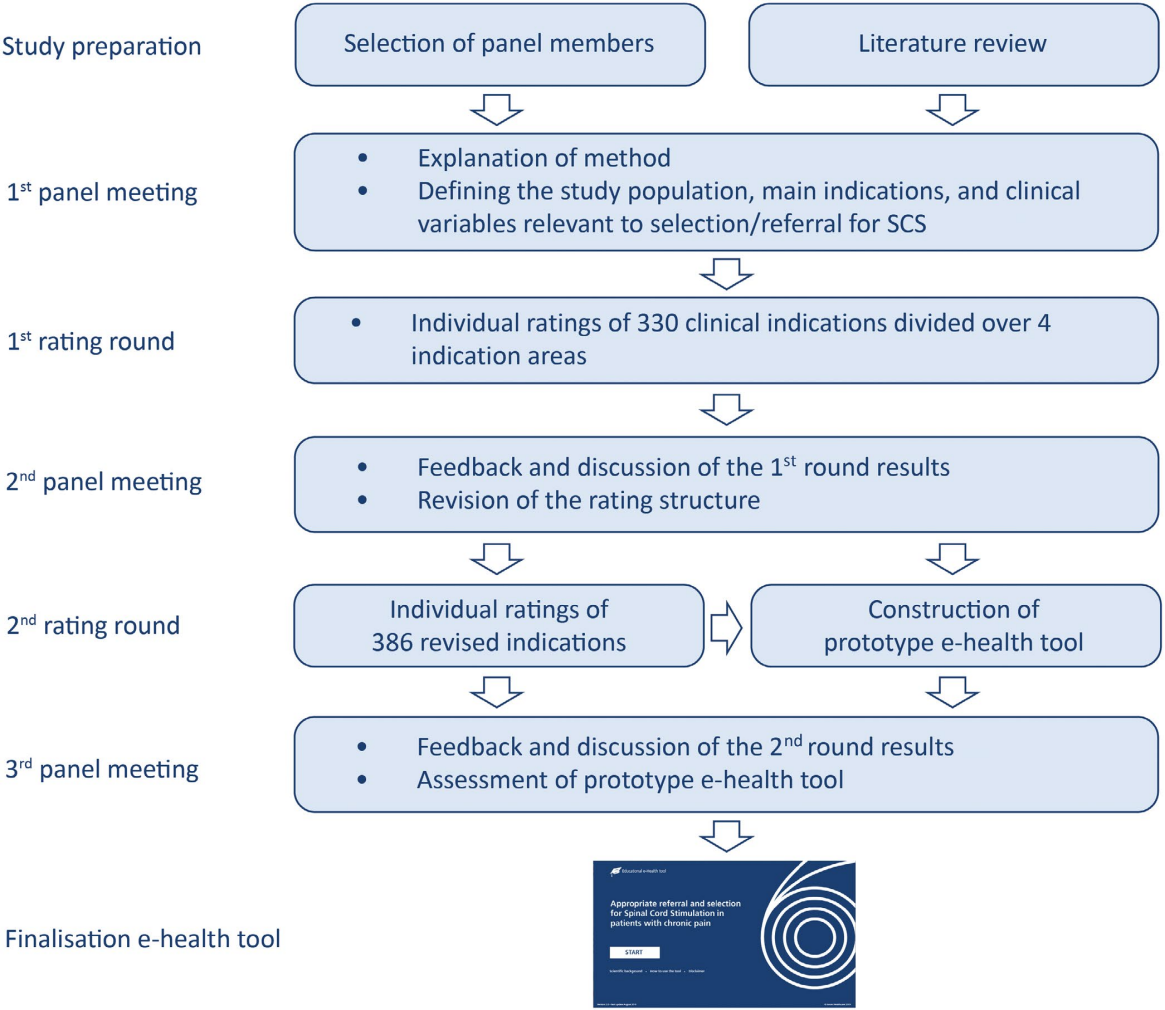
Flow diagram of the panel study

The flowchart of the panel process is shown in Figure 1. During the first panel meeting (December 2018; Chantilly, France) the panel reviewed the results of the literature study and formulated the starting points for the study. Selected inclusion and exclusion criteria for the consideration of SCS are listed in Table 1. The panel further identified four key indication areas for SCS in chronic pain: (a) chronic low back and leg pain (CBLP), (b) CRPS, (c) NPS (such as mono‐ and polyneuropathies) and 4) Ischaemic Pain Syndromes (IPS). For each of these indication areas the panel identified specific clinical variables that may be relevant for the decision of (referral for) SCS. These included treatment history, type/nature and location of pain, anatomic abnormalities, response to previous procedures (e.g. root block, transcutaneous electrical nerve stimulation [TENS] and/or medication) and spread of pain.

**TABLE 1:** Absolute criteria for the consideration of SCS, selected by the expert panel

Inclusion criteria	Exclusion criteria
Age ≥ 18 years Chronic pain with a duration of least 6 months One of the following primary indications: Chronic low back/leg pain Complex regional pain syndrome Neuropathic pain syndrome Ischaemic pain syndrome Pain severity at least moderate (VAS ≥ 5) having a substantial impact on daily functioning and quality of life Insufficiently responding to appropriate trials of medication and/or minimally invasive treatments (such as local anaesthetic nerve blocks) and/or experiencing intolerable side effects of these treatments No clear benefits of surgery expected	Unwilling to have an implant Unable to manage the device Absolute contra‐indications for active treatment (e.g. unfit for undergoing SCS, pregnancy, spine infection, coagulation disorder) Uncontrolled disruptive psychological or psychiatric disorder Ongoing alcohol and drug misuse Widespread pain

Based on these indication areas and variables, a set of 330 clinical scenarios was compiled. Using an online rating program, the panel members were asked to individually assess the appropriateness of (referral for) SCS for all scenarios. For SCS, no distinction was made between the various types of SCS or related therapies (e.g. conventional, burst, HF10, dorsal root ganglion [DRG] stimulation). In addition, no distinction was made between the decision for a trial or direct implant, as it was assumed that these decisions are the competence of the implant centres. Furthermore, variation in national guidelines and health authority requirements prevents global recommendations in this respect. A 9‐point scale was used to express the appropriateness of (referral for) SCS (reference values: 1 = inappropriate, 5 = equivocal/uncertain and 9 = appropriate). Panellists were instructed to consider only the clinical perspective, and to disregard cost of treatment, reimbursement environment and other potential constraints.

The panel discussed the results of the first rating round during a second meeting (Amsterdam, March 2019). This led to a number of adaptations and refinements of the clinical scenarios and related definitions.

The definitions of neuropathic and nociceptive pain received much attention. Recently, the International Association for the Study of Pain (IASP, 2019) defined the term “nociplastic” pain as “pain that arises from altered nociception despite no clear evidence of actual or threatened tissue damage causing the activation of peripheral nociceptors or evidence for disease or lesion of the somatosensory system causing the pain”. Besides nociceptive and neuropathic pain, this is a third type of pain descriptor. However, as this term was just recently proposed and officially accepted, it is not commonly known and may be confusing for non‐(pain) specialists. The panel therefore elected to replace this term with “neuropathic‐like pain” adding “without sensory disturbances”.

The final set comprised 386 scenarios (Table 2) for which the appropriateness of (referral for) SCS was assessed during the second individual rating round (May 2019). In addition to the clinical variables, the panel selected a set of psychosocial factors that may reduce the effectiveness of SCS and should therefore be included in the considerations for referral and selection for SCS. These factors were derived from scrutinizing the available literature, and a group discussion on expert panel members’ observations from clinical practice. The shortlist of psychosocial factors included: lack of engagement, dysfunctional coping, unrealistic expectations, inadequate daily activity level, problematic social support, secondary gain, psychological distress/mental health problems and unwillingness to reduce high‐dose opioids (Beltrutti et al., 2004; Blackburn et al., 2016; Bruns & Disorbio, 2009; Celestin, Edwards, & Jamison, 2009; Doleys, 2006; van Dorsten, 2006; Gybels et al., 1998; Paroli et al., 2018; Rosenberg, Schultz, Duarte, Rosen, & Raza, 2014; Shamji, Rodriguez, Shcharinsky, & Paul, 2016; Sparkes et al., 2010).

**TABLE 2:** Variables used to create the clinical scenarios (2nd round)

Chapter	Variables	Categories
CBLP	1. Previous spine surgery	No; yes
	2. Dominant location of pain	Leg; back; mixed
	3. Dominant type of pain	Neuropathic (with sensory disturbances); neuropathic‐like (without sensory disturbances); nociceptive; mixed
	4. Anatomic abnormality	Recurrent disc; scar tissue; iatrogenic nerve lesion; spinal/foraminal stenosis; spinal instability; none or not concordant with symptoms
	5. Response to root block, TENS, epiduroscopy, radiofrequency and/or neuropathic pain medication	No clinically relevant response; at least partial/temporary effect to any of these regimens
CRPS	1. Dominant symptom	Neuropathic pain (with sensory disturbances); neuropathic‐like pain (without sensory disturbances); ischaemic pain/vasomotor disturbance; “pure” nociceptive pain; mixed
	2. Response to sympathetic nerve block and/or neuropathic pain medication	No clinically relevant response; at least partial/temporary effect to any of these regimens
	3. Spread of pain	1 limb; 2 upper or 2 lower limbs; 1 upper and 1 lower limb; 3 or more limbs
NPS	1. Nature/origin of pain	Diabetic peripheral neuropathy; traumatic nerve lesion(s); post‐surgical pain; post‐herpetic pain; phantom pain; stump pain; brachial plexus injury without root avulsion; brachial plexus injury with root avulsion; small fibre neuropathy; post‐chemotherapy neuropathy
	2. Dominant type of pain	Neuropathic; nociceptive; mixed
	3. Response to TENS, somatic sensory and/or autonomic nerve block and/or neuropathic pain medication	No clinically relevant response; at least partial/temporary effect to any of these regimens
	4. Spread of pain	Both legs affected; both legs and arms affected; mononeuritis only; not applicable
IPS	1. Nature/origin of pain	Refractory angina pectoris; ischaemic leg pain (Fontaine II‐III); ischaemic leg pain (Fontaine IV); Raynaud’s disease; Buerger’s disease
	2. Response to sympathetic nerve block, TENS and/or neuropathic pain medication	No clinically relevant response; at least partial/temporary effect to any of these regimens

Abbreviations: CBLP, chronic low back/leg pain; CRPS, complex regional pain syndrome; IPS, ischaemic pain syndrome; NPS, neuropathic pain syndrome.

### 2.4 Appropriateness calculations and statistical analysis

Appropriateness of (referral for) SCS was calculated using the mathematical rules that are typically applied in RUAM studies (Fitch et al., 2001). SCS was considered appropriate if the median score was between 7 and 9, and inappropriate if the median was between 1 and 3, without disagreement between panellists. Disagreement was defined as the situation in which at least one third of the panellists scored in each of the sections 1–3 and 7–9 (Fitch et al., 2001). All other outcomes were deemed “equivocal/uncertain”. Frequency tables and cross‐tabulations were used to describe the appropriateness outcomes by clinical variables and specialty. All statistical analyses were performed using the IBM SPSS Statistics 26 software package (SPSS Inc.).

### 2.5 Educational e‐health tool

The panel results were used to develop an e‐health tool that supports the education of healthcare professionals on the appropriate referral and selection of patients with chronic pain for SCS. The tool combines the patient's clinical profile and psychosocial factors to an overall perspective on the appropriateness of (referral for) SCS. Details on the construction of the e‐health tool are provided in the supporting materials. The tool was presented and pretested during various workshops (a.o. the biennial EFIC congress [Valencia, September 2019] and the NSUKI Annual Scientific Meeting [Leeds, November 2019]). Based on the feedback from these meetings, further refinements were made to the tool.

## 3 RESULTS

### 3.1 Appropriateness and agreement

**FIGURE 2:**
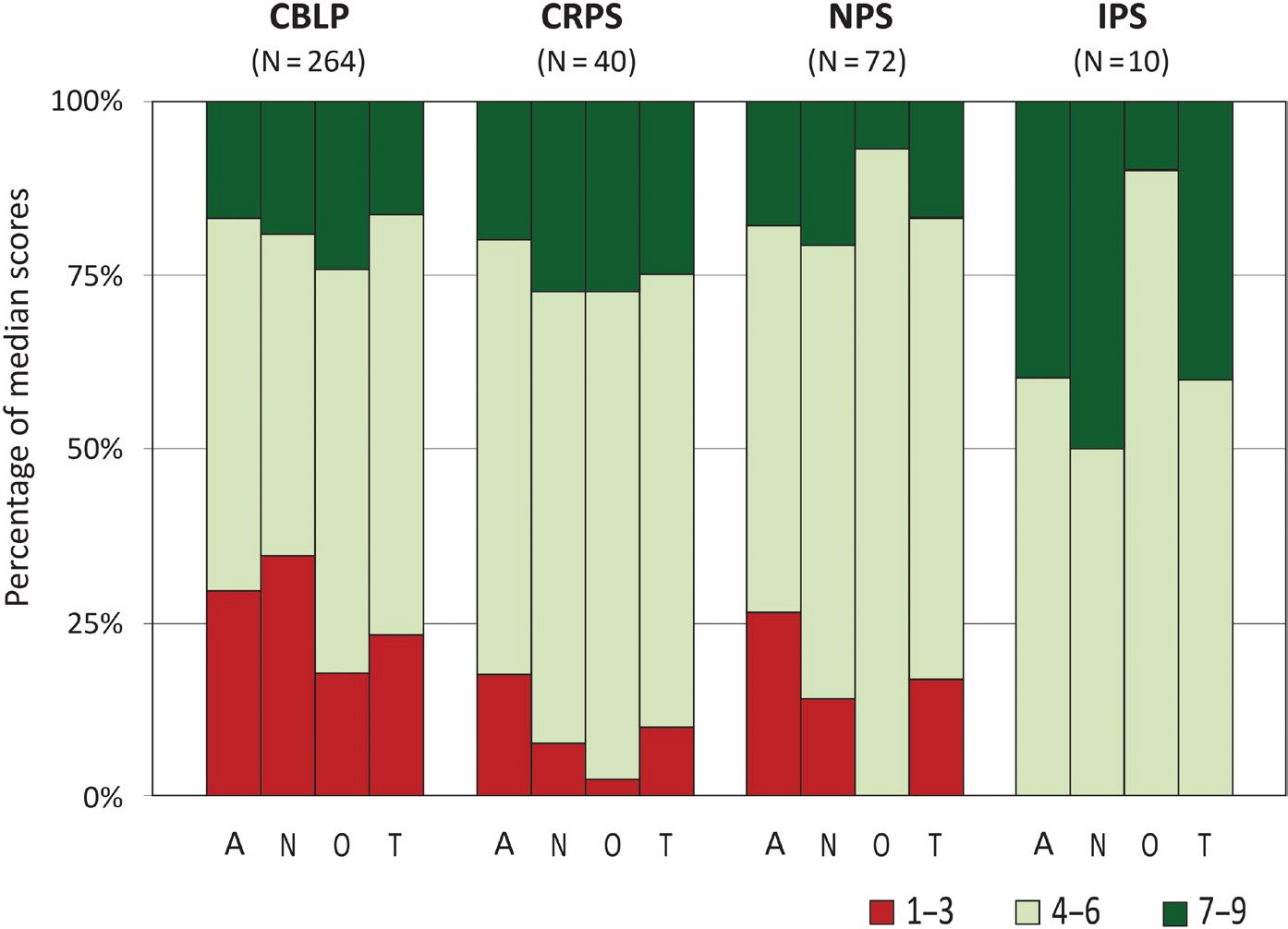
Appropriateness results by indication area and specialty of the panel members. Percentages of median scores in each of the sections 1–3 (inappropriate), 4–6 (equivocal) and 7–9 (appropriate). CBLP, chronic low back/leg pain; CRPS, complex regional pain syndrome; IPS, ischaemic pain syndrome; NPS, neuropathic pain syndrome. A, anaesthesiologists; N, neurosurgeons; O, other specialists (psychologist, physiotherapist, nurse specialist); T, total

Overall, (referral for) SCS was considered appropriate in 16% of the clinical scenarios, and inappropriate in 23%. For the remaining 61%, the outcome was equivocal. Appropriateness figures by indication area and specialty are depicted in Figure 2.

Chronic low back and leg pain, CRPS and NPS show fairly similar appropriateness patterns, while for the small chapter of IPS (10 scenarios) no inappropriate indications are seen. The category “other specialists” shows the highest proportion of equivocal outcomes for all indication areas. There was full agreement (median scores in the same appropriateness category) between the three specialities in 47% of the scenarios.

### 3.2 Chronic low back and/or leg pain

Table 3 shows the appropriateness of (referral for) SCS for patients with CBLP by clinical variables. In the case of previous spinal surgery, appropriateness was higher if leg pain was more dominant, if the neuropathic component of the pain was larger and if there has been at least a partial or temporary response to a root block, TENS, radiofrequency and/or neuropathic medication in the past. For anatomic abnormality, the highest appropriateness scores were seen for scar tissue and iatrogenic nerve lesion. For cases without previous spinal surgery, the patterns for the clinical variables were similar, but appropriateness figures were consistently and proportionally lower. The impact of the positive factors was mostly cumulative. For example, for patients with previous surgery and presenting with predominant leg pain, appropriateness goes up from 39% to 58% if the type of pain is predominantly neuropathic, and further increases to 83% if there had been a positive response to a root block. The patterns for inappropriateness largely mirrored those of the figures for appropriate outcomes.

**TABLE 3:** Appropriateness of (referral for) SCS by clinical variables for patients with chronic low back and/or leg pain. Appropriate indications; percentage of clinical scenarios by subgroup. Row totals per variable are 100%

Variables/categories	Previous surgery (%)			No previous surgery (%)		
	I	E	A	I	E	A
Dominant location of pain						
Leg	0	61	39	3	75	22
Mixed	23	48	29	25	60	15
Back	38	62	0	40	60	0
Dominant type of pain						
Neuropathic	8	53	39	8	67	25
Neuropathic‐like	3	64	33	11	72	17
Mixed	11	83	6	12	88	0
Nociceptive	88	12	0	88	12	0
Response to root block, TENS, RF and/or neuropathic pain medication						
No	27	59	14	32	65	3
At least partial or temporary	17	54	29	17	63	20
Anatomic abnormality						
Recurrent disc	14	63	23	14	72	14
Scar tissue	14	45	41	14	63	23
Iatrogenic nerve lesion	14	45	41	14	63	23
Spinal/foraminal stenosis	18	64	18	18	77	5
Spinal instability	50	50	0	59	41	0
None/not concordant with symptoms	23	72	5	27	68	5

Abbreviations: A, appropriate; E, equivocal; I, inappropriate.

### 3.3 Complex regional pain syndrome

For patients with CRPS, (referral for) SCS was considered more frequently appropriate if the type of pain was neuropathic(‐like) or ischaemic due to vasomotor disturbances, and if spread of pain was more limited (Table 4). In addition, there was a pronounced impact of a positive response to a previous nerve block and/or neuropathic medication on the perceived appropriateness of SCS. Inappropriateness was mainly seen in patients with nociceptive or widespread pain.

**TABLE 4:** Appropriateness by clinical variables for patients with complex regional pain syndrome. Percentage of clinical scenarios by variable. Row totals are 100%

Variables/categories	Inappropriate (%)	Equivocal (%)	Appropriate (%)
Dominant symptom			
Neuropathic	0	50	50
Neuropathic‐like	0	75	25
Ischaemic/vasomotor	0	62	38
Mixed	12	75	13
Nociceptive	38	62	0
Response to nerve block and/or neuropathic pain medication			
No	15	80	5
At least partial or temporary	5	50	45
Spread of pain			
One limb	0	50	50
Two upper or two lower limbs	10	70	20
One upper and one lower limb	0	70	30
Three or more limbs	30	70	0

### 3.4 Neuropathic pain syndromes

Of the 10 indications considered by the panel, traumatic nerve lesions and postsurgical pain had the highest appropriateness scores (both 33% of related scenarios), while postherpetic pain and brachial plexus injury with root avulsion were never considered an appropriate indication (mostly equivocal). For the other 6 indications, 17% of related scenarios were considered appropriate. The presence of predominant neuropathic pain significantly increased the perceived appropriateness of SCS (overall from 17% to 50%), while the addition of a previous positive response to a nerve block or neuropathic medication further increased this figure to 75%. Details can be found in the supporting materials.

### 3.5 Ischaemic pain syndromes

Refractory angina pectoris, ischaemic leg pain (Fontaine II‐III), Raynaud's disease and Buerger's disease were all considered an appropriate indication for SCS provided a positive response to a previous nerve block or neuropathic pain medication. In the case of no response to a previous block or medication, and for patients with ischaemic leg pain categorized as Fontaine IV, the outcome was equivocal.

### 3.6 Psychosocial factors

After preparatory work by the psychologist panellists, the panel agreed on a set of factors and related definitions that should guide the psychosocial evaluation of patients for whom SCS is considered an option (Table 5). Recommendations were specified by target group (referrers/implanters). For referrers, classification in any of the moderate or severe categories (or comparable) should lead to consultation with a clinical psychologist or multidisciplinary team. For implanters, similar advice applied to the moderate category, but any outcome in the severe category was considered a strong contraindication for SCS, assuming that this situation has been determined by a standardized measurement or specialist clinical judgement. For both groups, a total lack of engagement was considered an absolute contraindication.

**TABLE 5:** Psychosocial factors judged relevant for the consideration of SCS in patients with chronic pain

Variables/categories and related definitions
*Lack of engagement* (no; partly; total)
Failing to attend appointments (offered by the neuromodulation or other services)
Failing to follow up on agreed recommendations, e.g. self‐referral to psychological therapy service
Non‐compliance with treatment
Attending treatment (e.g. a pain management programme) but with a clear lack of engagement with the programme (e.g. frequently attending late, non‐participation in group tasks, not completing homework tasks/exercise programme)
*Dysfunctional coping* (no or mild; moderate; severe)
Avoidance of movement/activity
Avoidance, misuse of medication/illegal drugs
*Unrealistic expectations* (no or mild; moderate; severe)
Total pain relief
Inability to articulate post‐implant goals
*Inadequate daily activity level* (no or mild; moderate; severe)
Inconsistency between what patient reports they can do and what they have shown they can do, e.g. patient reporting they cannot get out of bed for short period but attends all appointments
Low self‐efficacy
Lack of, or very restricted, participation in activities of daily living
Problematic social support (no or mild; moderate; severe)
No social/family support
Poor quality support, e.g. patient reports they have friends/family but are unreliable, patient has not sought their support
Secondary gain (no; probably; yes or very likely)
Litigation
Presence of factors that mean that the patient might (unconsciously) have an incentive for remaining “ill”
*Psychological distress/mental health problems* (no or mild; moderate; severe)
For example: low mood, anxiety, panic disorder, post‐traumatic stress disorder
*Unwilling to reduce high‐dose opioids* (no, or not applicable; probably; very likely)
Use of high‐dose opioids, and unwilling to reduce these to an acceptable level according to the opinion of the treating physician

### 3.7 Educational e‐health tool

**FIGURE 3:**
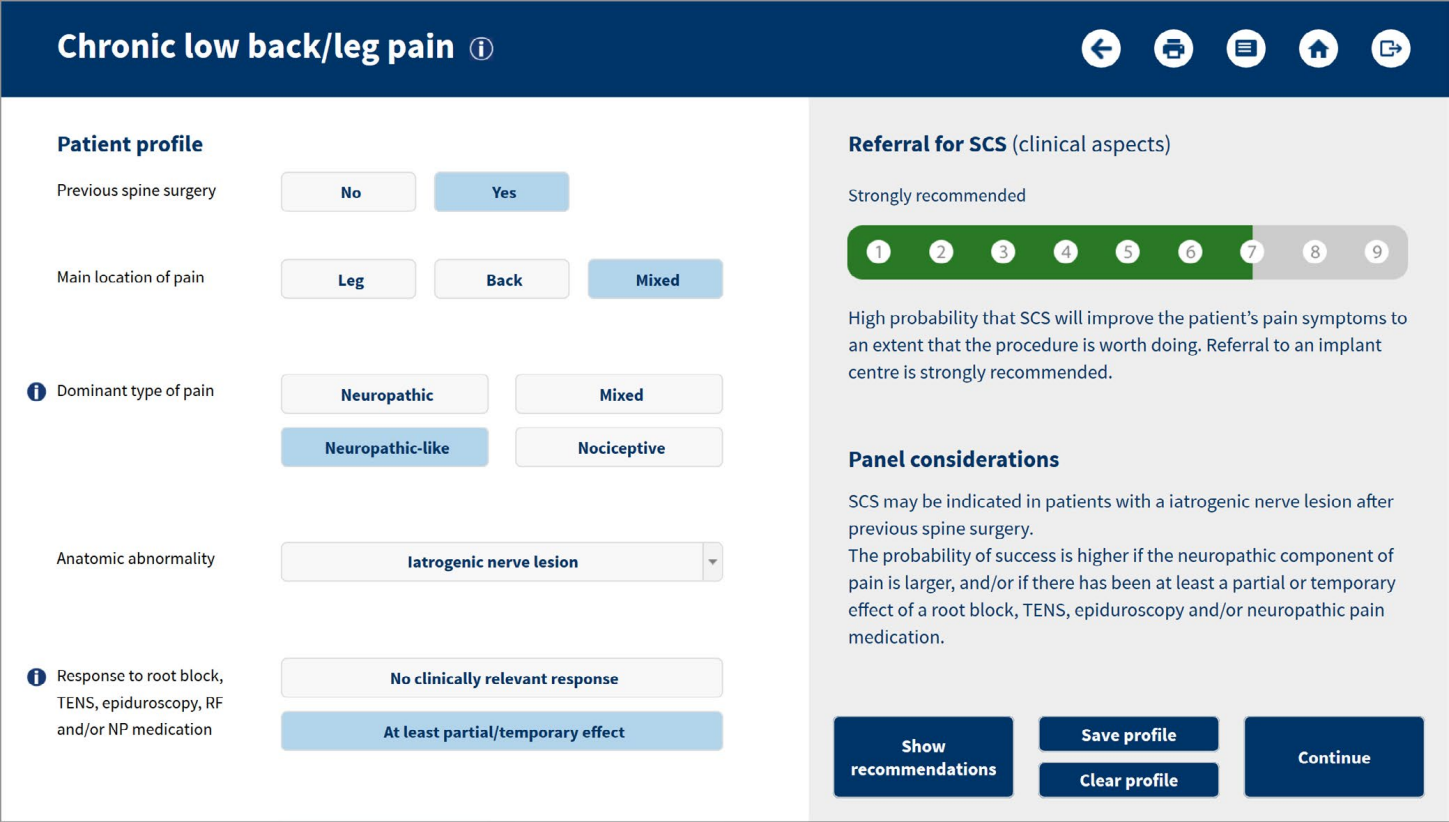
User interface of the e‐health tool (clinical aspects)

**FIGURE 4:**
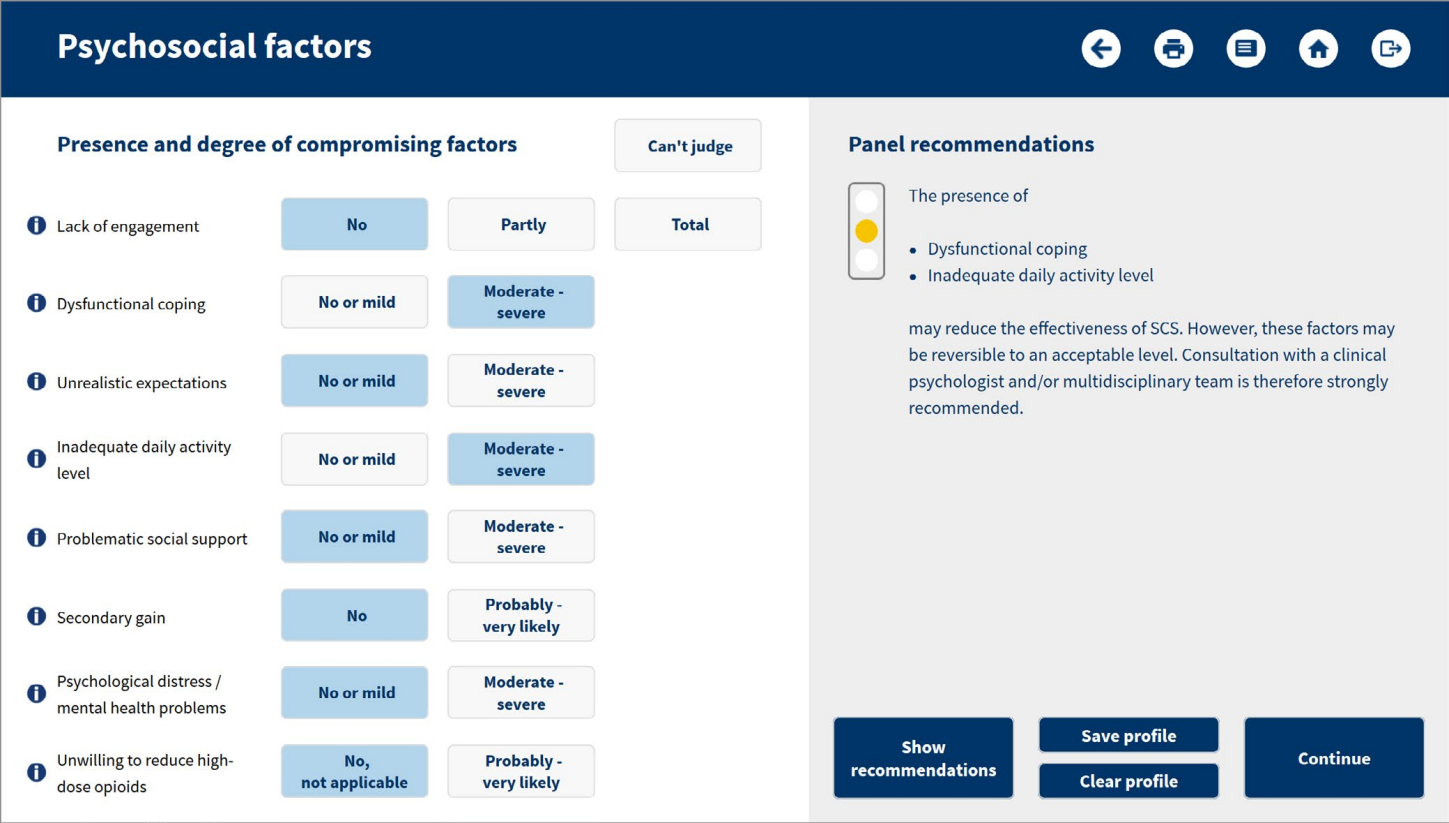
User interface of the e‐health tool (psychosocial factors)

The panel results were embedded in an educational e‐health tool that allows creating a patient profile and seeing the appropriateness of (referral for) SCS. The tool starts with a check on the absolute criteria for the consideration of SCS (see Table 1), and continues with an assessment of the clinical aspects (see Figure 3). If the outcome is appropriate or equivocal, the psychosocial profile needs to be completed (Figure 4). The combined outcomes of the clinical and psychosocial aspects result in a final recommendation (referral/SCS strongly recommended, recommended or not recommended). For details on the calculations: see supporting materials.

The tool was presented and tested during workshops at congress meetings of EFIC and NSUKI, as well as some other (inter)national educational meetings, where it was received with significant interest and even enthusiasm. Based on the feedback of participants, some adaptations and refinements were made to the tool.

The e‐health tool is freely available for healthcare professionals on: https://www.scstool.org/.

## 4 DISCUSSION

Although SCS has been used for 50 years in the management of chronic pain, there remain a number of uncertainties about its precise indications in daily clinical practice. This is partly due to a lack of high(er)‐quality studies into the effectiveness of SCS for some indication areas, but also to the heterogeneity of the patient population within the largest indications of SCS such as neuropathic pain and persistent pain after spine surgery. Finally, the interaction between chronic pain and psychosocial factors as well as analgesic medication intake provide further layers of complexity. This in turn necessitates careful balancing of relevant clinical and psychosocial characteristics.

The lack of high(er)‐quality effectiveness studies especially relates to the mono‐ and polyneuropathies such as postsurgical and postherpetic pain, with the exception of painful diabetic polyneuropathy (van Beek et al., 2018; Slangen et al., 2014; de Vos et al., 2014). Efficacy of SCS in ischaemic pain syndromes has been shown in older studies, but considerable bias downgrades the quality of evidence and makes these outcomes uncertain (Huygen et al., 2019).

The RUAM proved to be helpful in establishing patient‐specific appropriateness statements for 386 clinical scenarios. Despite the differences in panellists’ background, there was substantial agreement across the specialties (Figure 2). The panel outcomes constitute clear situations in which SCS is appropriate or inappropriate. However, there were also a considerable number of scenarios for which the outcome was equivocal, reflecting uncertainty about the potential effectiveness of SCS. It should be mentioned that the appropriateness figures relate to a set of theoretical scenarios, and part of the ratings may be equivocal due to uncommon or less realistic cases. As demonstrated in previous RUAM studies, the proportion of equivocal/uncertain outcomes may therefore be lower in real clinical practice (e.g. Anselmetti et al., 2013; Schupfner et al., 2016).

### 4.1 Clinical factors

For all pain areas, a partial or temporary response to a previous root block, TENS and/or neuropathic pain medication consistently increased the appropriateness ratings for SCS. This is not directly supported by literature findings, but obviously reflects the panellists’ experiences in clinical practice. A second strong driver of appropriateness (except for IPS) was the neuropathic(‐like) pain component. This is in line with the inclusion criteria for most clinical trials that took predominant neuropathic(‐like) pain as a prerequisite for the consideration of SCS.

In our study, neuropathic pain (with sensory disturbances) generally scored higher than neuropathic‐like pain (without sensory disturbances) as a predictor of suitability for neurostimulation. Assuming that in the majority of patients with CBLP and CRPS there is more neuropathic‐like pain than neuropathic pain, this is a remarkable finding. After all, there is good‐quality evidence that neurostimulation is effective for both indications. The different outcomes for neuropathic and neuropathic‐like pain may partly be explained by unfamiliarity with the various definitions by the panel members, and we are therefore hesitant to emphasize this difference too much. At the same time, we advocate explicitly recognizing this difference because we want to emphasize that the indication for neurostimulation is not limited to neuropathic pain alone.

In patients with CBLP, the appropriateness scores were higher in those who had undergone spinal surgery. This is not surprising, as the efficacy of SCS in CBLP has predominantly been studied in patients with “failed back surgery syndrome”. However, a recent cohort study on high‐frequency SCS in surgery‐naïve patients suggests favourable results (Al‐Kaisy et al., 2018; Kapural et al., 2015).

The appropriateness of SCS was considered higher when the pain was located more in the leg than in the back. This perception also relates to the design of several RCTs, where patients with predominant back pain were excluded (Kumar et al., 2007; North et al., 2005). However, a meta‐analysis stratified by the location of CBLP pain did not find a statistical association between pain relief and the location of CBLP (Taylor, Desai, Rigoard, & Taylor, 2014).

The highest appropriateness scores were given to patients with scar tissue or iatrogenic nerve lesions, albeit that this cannot be supported by data from clinical studies or guideline recommendations. It seems worthwhile to explore this general feeling with controlled well‐powered studies. The panel outcomes for surgery‐naïve patients with CBLP are largely in line with the results of a recent RUAM study into the appropriate management of patients with persistent pain after previous spine surgery (Tronnier et al., 2019).

For patients suffering from CRPS, spread of pain is an important variable, with the highest appropriateness score given to patients with pain limited to one extremity. Most current guidelines and literature do not mention the impact of spread of pain on the efficacy of SCS. However, the two trials that have been conducted on SCS in CRPS included only patients with pain limited to one hand or one foot (Kemler et al., 2000), or to the lower limbs (Deer et al., 2017), indicating that the perceived relation between limited spread of pain and efficacy of SCS already existed in the pain specialist community.

For (other) NPS, the outcomes were mostly equivocal, tending to appropriate in patients with predominant neuropathic pain and having had a temporary/partial response to a previous nerve block, TENS and/or neuropathic pain medication, and to inappropriate in the opposite scenarios. Although these appropriateness patterns were fairly straightforward, there is, as yet, no direct evidence from clinical trials to support the panel recommendations. In the absence of evidence from clinical trials, we can sometimes use a more mechanism‐based approach in decision making. It is, for example, generally accepted that in the case of nerve damage, stimulation is only effective if applied proximal to the lesion. This is why a brachial plexus injury with root avulsion and other neuropathic conditions proximal to the DRG cannot be considered an appropriate indication (De Andrade et al., 2010; Sdrulla, Guan, & Raja, 2018; Sindou, Mertens, Bendavid, García‐Larrea, & Mauguière, 2003).

For IPS, the expert panel indicated that SCS should always be considered an option, as the outcomes were never inappropriate. It should be noted that the panel was asked to assess the appropriateness of SCS in relation to pain reduction, and not to improve blood flow or reduce the risk of limb amputation. The panel's positive attitude towards SCS in IPS is corroborated by data from literature, reviewed by Ubbink and Vermeulen (2013). The panel outcomes for patients with ischaemic leg pain classified as Fontaine IV (equivocal) may be surprising as this condition is associated with ischaemic ulcers or gangrene for which SCS may seem less appropriate. However, data from a single‐arm study suggest that SCS in patients with Fontaine III‐IV lower limb critical ischemia may result in significant pain relief (Petrakis & Sciaccia, 2000).

### 4.2 Psychosocial factors

The panel selected and defined a set of eight psychosocial factors that are relevant when considering referral or selection for SCS. These factors were based on the (fragmentary) evidence from studies (Beltrutti et al., 2004; Blackburn et al., 2016; Bruns & Disorbio, 2009; Celestin et al., 2009; Doleys, 2006; van Dorsten, 2006; Gybels et al., 1998; Paroli et al., 2018; Rosenberg et al., 2014; Shamji et al., 2016; Sparkes et al., 2010) and expert panel members’ observations from clinical practice. The current checklist and related recommendations should be seen as a first step in the systematic evaluation of psychosocial aspects by nonexperts. In this phase, simplicity and (time)efficiency of the checklist have prevailed over comprehensiveness of the aspects included.

### 4.3 Strengths and limitations

Our study has produced a set of patient‐specific recommendations on the appropriateness of SCS, combining relevant clinical and psychosocial aspects. The inclusion of these recommendations in an easy‐to‐use educational e‐health tool may help to improve the quality of (pre)selection of patients with chronic pain for SCS.

The most important limitations of this study are related to the panel composition and the subjective nature of the panel recommendations. We have included only healthcare professionals who are actively involved in patient selection for SCS. We have chosen this approach because we considered practice experience as a prerequisite to assess the appropriateness of SCS for the various scenarios. However, for assessing the applicability of the panel recommendations in daily practice, the involvement of referring physicians will be absolutely necessary.

The absence of results from clinical studies for many of the clinical scenarios means that expert opinion was often the best available evidence. Validation studies are therefore mandatory, and have already been initiated. RUAM studies in other disease areas have demonstrated good predictive value of the panel recommendations for eventual patient outcomes (Garcia‐Gutierrez et al., 2014; Gimeno García et al., 2012; Lawson et al., 2012; Sekhri et al., 2008; Tombal et al., 2013).

Finally, the list of (potential) indications for SCS in chronic pain we have considered in this study is not exhaustive. We have concentrated on the most prevalent indications, and other conditions may be added by the panel in future updates.

### 4.4 Conclusions

Using the RUAM, a European multidisciplinary panel formulated patient‐specific recommendations on the appropriate referral and selection of patients with chronic pain for the consideration of SCS. The e‐health tool may help physicians learn to apply an integrated approach of clinical and psychosocial factors.

The predictive value of the panel recommendations for patient outcomes needs further research.

## CONFLICTS OF INTEREST

All panellists, except H.B., were financially compensated for their time and reimbursed for travel and hotel costs. Additional disclosures: S.T.: consultancy: Boston Scientific, Mainstay Medical. F.H.: advisory board: Abbott, Saluda, Salvia, Pfizer, Boston Scientific. S.P.: consultancy/advisory board: Boston Scientific. JDA: consultancy (education/training): Medtronic, Boston Scientific; research grant: Boston Scientific; advisory board: Boston Scientific. G.B.: advisory board: Abbott, Boston Scientific, Nevro, Nalu; research grant: Abbott, Nevro; shareholder: Nalu. H.B.: nothing to declare. N.B.: nothing to declare. B.B.: consultancy: Abbott, Stim Wave, Medtronic, Nevro, Salvia Bioelectronics. J.C.: nothing to declare. G.D.C.: nothing to declare. L.D.: Consultant (speaker): Boston Scientific, Abbott, Sandoz, Grunenthal. S.E.: personal consulting: Medtronic, Abbott, Saluda Medical, Mainstay Medical; departmental: Medtronic, Nevro. K.G.: consultancy (teaching/training): Medtronic, Boston Scientific, Abbott, Nevro; advisory boards: Medtronic, Boston Scientific. J.W.K.: advisory board: Boston Scientific, Abbott, Saluda. K.M.: teaching fees/travel support: Abbott, Medtronic. M.P.: nothing to declare. A.S.: nothing to declare. M.W.: Speaker fees: Boston Scientific, St. Jude Medical (Abbott), Medtronic. H.S.: honoraria from Boston Scientific for advice to the design of the study and data analysis.

## AUTHOR CONTRIBUTIONS

S.T. was the principal investigator, helped to obtain funding and chaired the panel meetings. S.T., F.H. and H.S. designed the study. H.S. advised on the methodology, performed the statistical analyses and supervised the construction of the electronic tool. S.T., F.H., S.P. and H.S. prepared the draft manuscript. All other authors participated in the panel meetings, performed the appropriateness ratings and assisted in the interpretation of data. All authors have reviewed and approved the final version of the manuscript.

## Supporting information

Supplementary Material
