# Supplementary material for: Appropriate referral and selection of patients with chronic pain for spinal cord stimulation: European consensus recommendations and e‐health tool
Source: Eur J Pain. 2020 Apr 4;24(6):1169–81. doi: 10.1002/ejp.1562 (PMC7318692; doi:10.1002/ejp.1562)
Supplement: Supplementary file 1 — Supplementary Material [file EJP-24-1169-s001.docx]

**SUPPORTING INFORMATION: PANEL MEMBERS**

**Anaesthesiologists**

- José De Andrés (Valencia, Spain)
- Ganesan Baranidharan (Leeds, United Kingdom)
- Bart Billet (Roeselare, Belgium)
- Giuliano De Carolis (Pisa, Italy)
- Laura Demartini (Pavia, Italy)
- Sam Eldabe (Middlesbrough, United Kingdom)
- Frank Huygen (Rotterdam, The Netherlands)
- Jan Willem Kallewaard (Arnhem, The Netherlands)
- Kaare Meier (Aarhus, Denmark)
- Simon Thomson (Basildon, United Kingdom)

**Clinical nurse specialist**

- Angela Stark (Basildon, United Kingdom)

**Clinical psychologists**

- Neil Berry (Southampton, United Kingdom)
- Mery Paroli (Pisa, Italy)
- Simon Prangnell (Oxford, United Kingdom)

**Neurosurgeons**

- Hayat Belaid (Paris, France)
- Kliment Gatzinsky (Gothenburg, Sweden)
- Matthias Winkelmüller (Hannover, Germany)

**Physiotherapist**

- Jan Cooil (Basildon, United Kingdom)

**SUPPORTING INFORMATION: CONSTRUCTION E-HEALTH TOOL**

Fig. S1 shows how the educational e-health tool combines the patient’s clinical profile (appropriateness ratings) and psychosocial factors to an overall recommendation on (referral for) SCS.


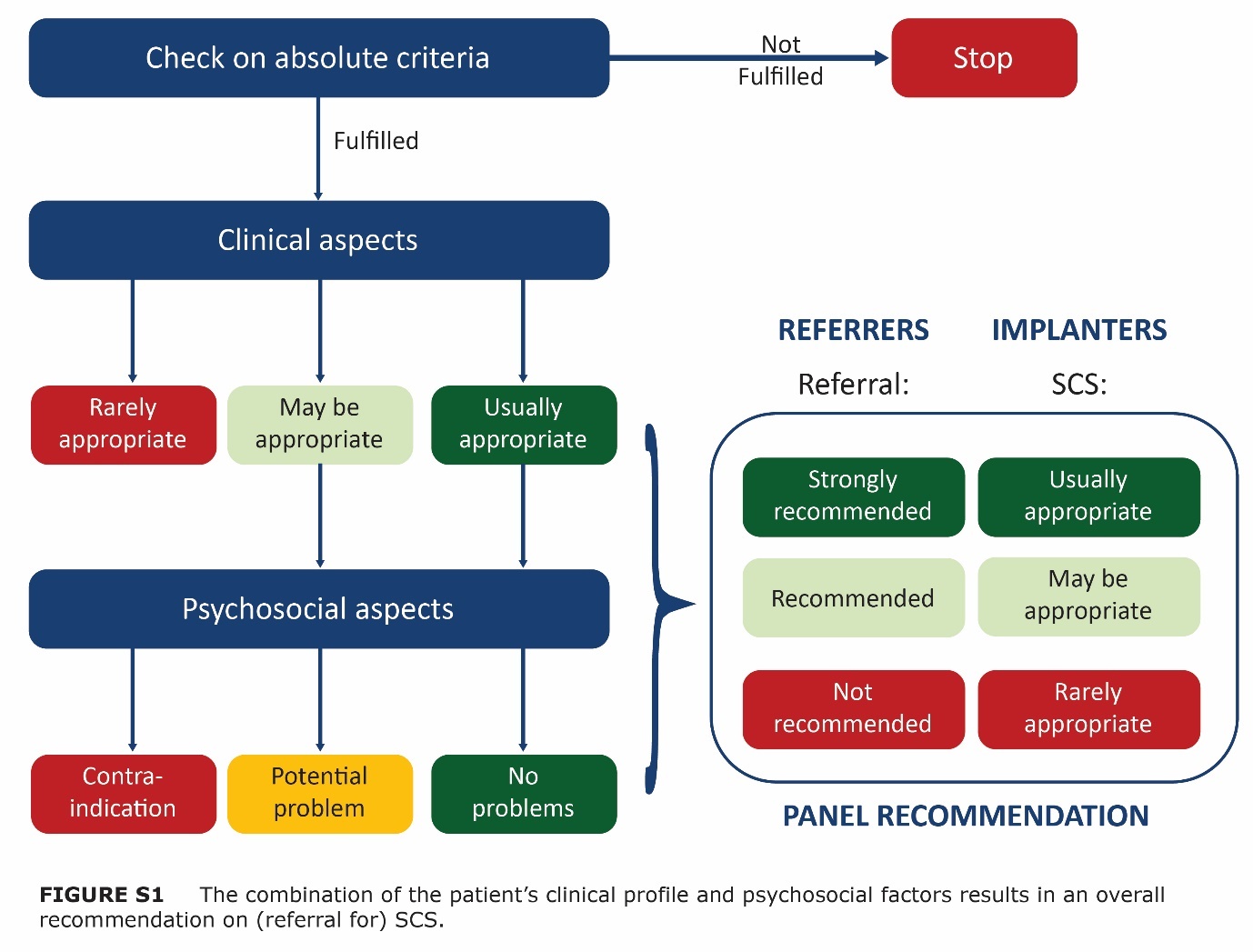


After checking the absolute criteria, firstly the clinical aspects are assessed. If the appropriateness outcome is “rarely appropriate”, then (referral for) SCS is not recommended. Otherwise, the user is led to the next step. i.e. assessment of psychosocial aspects.

For the 8 psychosocial factors, there are different user interfaces and algorithms for referrers and implanters.

For referrers, there are mostly 2 categories for each of the variables: no-mild and moderate-severe, or comparable wordings. If all aspects are ticked “no-mild”, the outcome is green (no problems). If any of the other categories is chosen, the outcome is orange, indicating that there are compromising factors that may reduce the effectiveness of SCS, necessitating further consultation with a clinical psychologist or a multidisciplinary team. The variable “Lack of engagement” has 3 categories (no, partly, total). A total lack of agreement is considered an absolute contraindication for SCS.

For implanters, there are 3 categories for all variables: no-mild, moderate and severe, or comparable wordings. Similar to the referrer algorithm, the outcome is green (no problems) if all aspects are ticked “no-mild”. If at least one of the categories is clicked “moderate”, and no “severe” selections have been made, then the outcome is orange (potential problems, but potentially reversible to an acceptable level), and further consultation with a clinical psychologist is advised. If for any of the variables the category “severe” is clicked, this is considered a strong contraindication for SCS, assuming that this situation has been determined by a standardised measurement or specialist clinical judgement.

The final recommendation combines the outcomes of the clinical and psychosocial assessments, where the lowest value is normative. For example, if the outcome is green (usually appropriate) for the clinical aspects and orange (potential problems) for the psychosocial aspects, the final recommendation is light green, meaning that SCS may be appropriate, but that consultation with a clinical psychologist or multidisciplinary team is necessary.

**SUPPORTING INFORMATION: TABLE S1**

**TABLE S1** Appropriateness by clinical variables for patients with neuropathic pain syndromes (NPS). Percentage of clinical scenarios by variable. Row totals are 100%.

| Variables/categories | Inappropriate | Equivocal | Appropriate |
| --- | --- | --- | --- |
| Nature/origin of pain   - Diabetic peripheral neuropathy - Traumatic nerve lesion(s) - Post-surgical pain - Post-herpetic pain - Phantom pain - Stump pain - Brachial plexus injury without root avulsion - Brachial plexus injury with root avulsion - Small fiber neuropathy - Post-chemotherapy neuropathy | %  11  17  17  17  17  17  17  33  17  17 | %  72  50  50  83  67  67  67  67  67  67 | %  17  33  33  0  17  17  17  0  17  17 |
| Type of pain   - Predominant neuropathic - Predominant nociceptive - Mixed | 0  50  0 | 50  50  100 | 50  0  0 |
| Response to TENS, somatic sensory and/or autonomic nerve block and/or neuropathic pain medication   - No - At least partial or temporary | 15  5 | 80  50 | 5  45 |
| Spread of pain (only diabetic neuropathy)   - Both legs affected - Both legs and arms affected - Mononeuritis only | 0  33  0 | 83  67  67 | 17  0  33 |
